# LIPUS inhibits inflammation and catabolism through the NF‐κB pathway in human degenerative nucleus pulposus cells

**DOI:** 10.1186/s13018-021-02739-1

**Published:** 2021-10-18

**Authors:** Weiwei Yi, Qing Chen, Chuan Liu, Kaiting Li, Bailong Tao, Guihua Tian, Lu Zhou, Xiaohong Li, Jieliang Shen, Bo Liu, Zhenming Hu, Dawu Wang, Dingqun Bai

**Affiliations:** 1 Department of Rehabilitation Medicine, The First Affiliated Hospital of Chongqing Medical University, No. 1 Shiyou Street, Yuzhong District, Chongqing, 400010 China; 2 Laboratory Research Center, The First Affiliated Hospital of Chongqing Medical University, No. 1 Shiyou Street, Yuzhong District, Chongqing, 400010 China; 3 Department of Radiology, The First Affiliated Hospital of Chongqing Medical University, No. 1 Shiyou Street, Yuzhong District, Chongqing, 400010 China; 4 Department of Orthopedics, The First Affiliated Hospital of Chongqing Medical University, No. 1 Shiyou Street, Yuzhong District, Chongqing, 400010 China

**Keywords:** Low-intensity pulsed ultrasound, NF-κB, Inflammation, Nucleus pulposus cells

## Abstract

**Background:**

Low-intensity pulsed ultrasound (LIPUS) is a safe and noninvasive rehabilitative physical therapy with anti-inflammatory effects. The current study investigated the effect of LIPUS on the inflammation of nucleus pulposus (NP) cells and its underlying mechanism.

**Methods:**

Human NP cells were acquired from lumbar disc herniation tissue samples and cultured for experiments. Human NP cells were treated with LPS and then exposed to LIPUS (15 mW/cm^2^, 30 mW/cm^2^ and 60 mW/cm^2^) for 20 min daily for 3 days to determine the appropriate intensity to inhibit the expression of the inflammatory factors TNF-α and IL-1β. The gene and protein expression of aggrecan, collagen II, MMP-3 and MMP-9 was measured by real‐time PCR and western blotting, respectively. The activity of the nuclear factor‐kappa B (NF‐κB) pathway was examined by western blotting and immunofluorescence. After pretreatment with the NF-κB inhibitor PDTC, the expression of TNF-α, IL-1β, MMP-3 and MMP-9 was measured by real‐time PCR.

**Results:**

LIPUS at intensities of 15 mW/cm^2^, 30 mW/cm^2^ and 60 mW/cm^2^ inhibited LPS-induced NP cell expression of the inflammatory factors TNF-α and IL-1β, especially at 30 mW/cm^2^. LIPUS significantly upregulated the gene and protein expression of aggrecan and collagen II and downregulated the gene and protein expression of MMP-3 and MMP-9 in LPS-induced NP cells. The NF‐κB signaling pathway was inhibited by LIPUS through inhibiting the protein expression of p-P65 and the translocation of P65 into the nucleus in LPS-induced NP cells. In addition, LIPUS had similar effects as the NF-κB inhibitor PDTC by inhibiting the NF-κB signaling pathway, inflammation and catabolism in LPS-induced human degenerative nucleus pulposus cells.

**Conclusion:**

LIPUS inhibited inflammation and catabolism through the NF‐κB pathway in human degenerative nucleus pulposus cells.

## Background

Intervertebral disc degeneration is the initial factor in spinal degenerative diseases and one of the main causes of low back pain, which has caused serious economic and social burdens [1]. The current treatment methods for intervertebral disc degeneration mainly include conservative treatment and surgical treatment. Conservative treatment is limited to short-term symptom relief, while surgical treatment is invasive and destroys the biomechanical integrity of the intervertebral disc to some extent, and there is a risk of recurrence of intervertebral disc degeneration in the later stage [2]. Therefore, it is necessary to find a long-term, safe, effective and noninvasive treatment.

Nucleus pulposus (NP) cells are the main component of the intervertebral disc and are essential for maintaining the overall biomechanical function of the intervertebral disc. Many studies have confirmed that an excessive inflammatory response in NP cells is an important cytological pathological feature of degenerative intervertebral disc tissues [3, 4]. Many studies have shown that proinflammatory factors such as TNF-α and IL-1β stimulate the expression of matrix metalloproteinase (MMP) and induce NP cell apoptosis, resulting in the loss of collagen and proteoglycans and leading to intervertebral disc degeneration [5, 6]. NF-κB is a multifunctional transcription factor that plays an important role in regulating the inflammatory response, cell proliferation and apoptosis. In particular, the NF-κB pathway has been reported to mediate catabolic and inflammatory processes in human degenerative NP cells [7, 8]. Jianwei Liu et al. showed that andrographolide inhibited the expression of degenerative and inflammatory mediators by inhibiting the NF-κB pathway [9]. Therefore, regulation of the NF‐κB pathway may play a key role in the inflammatory control and catabolism of NP cells.

Low-intensity pulsed ultrasound (LIPUS) is a commonly used rehabilitation treatment that has anti-inflammatory and anti-apoptotic properties. Because of its safety, noninvasiveness, effectiveness and convenient advantages, it is widely used in clinical practice including the treatment of soft tissue injury, tenosynovitis and osteoarthritis, the promotion of fracture healing and the treatment of nonunion [10–13]. However, studies on the relationship between LIPUS and intervertebral disc degeneration only show that LIPUS can promote the synthesis of NP cell extracellular matrix [14], but the role of LIPUS on inflammation in intervertebral disc degeneration and its molecular mechanism has not yet been clarified. Studies have suggested that LIPUS can inhibit the inflammatory response in osteoblasts induced by LPS [15]. LIPUS prevents acute kidney injury through anti-inflammatory and anti-apoptotic effects [16]. In addition, LIPUS inhibited the expression of inflammatory factors in hPDLCs by inhibiting the NF‐κB signaling pathway [17]. Therefore, we hypothesize that LIPUS can inhibit inflammation and catabolism in human degenerative nucleus pulposus cells through the NF-κB pathway.

## Material and methods

### NP sample source, isolation and culture

All the NP tissues were acquired from patients with lumbar disc herniation undergoing discectomy treatment. The degree of intervertebral disc was assessed according to the Pfirrmann classification system by MRI scans [18]. Samples from patients were grades III–V (Table 1). The study protocol was subject to approval by the medical ethics committee of Chongqing Medical University. Written informed consent was obtained from all patients of 8 volunteers aged 42–68 years for undergoing discectomy treatment.

**Table 1:** The information of specimens for nucleus pulposus cell isolation

ID	Gender	Age	Segment	Grade
1	Female	42	L4-5	IV
2	Male	56	L4-S1	IV
3	Male	45	L5-S1	III
4	Female	58	L4-S1	V
5	Male	61	L5-S1	IV
6	Female	68	L4-5	IV
7	Male	52	L5-S1	V
8	Male	62	L4-5	IV

The NP tissue samples were washed twice with PBS, digested in 0.25% trypsin solution for 20 min and then in 0.2% type II collagenase for 4 h. The isolated cells were filtered through a 200-μm filter and resuspended in DMEM/F12 medium with 20% FBS, at 37℃, 5% CO_2_ atmosphere for culture.

### Cells treatment

The LIPUS apparatus that delivered an ultrasound signal was supplied by the biomedical engineering lab of Chongqing Medical University. For the detection of relative inflammation, LPS (500 μg/mL) was added into the medium to induce human NP cells for 24 h. After LPS treatment, human NP cells were treated without or with LIPUS (frequency of 1.5 MHz, pulse repetition rate of 1 kHz and the on–off ratio of 20%), at various intensities (15, 30 and 60 mW/cm^2^) for 20 min a day for 3 days to find the appropriate intensity for subsequent study. The coupling gel was used between the transducer and cell plate to ensure optimal ultrasound exposure.

### Quantitative real-time polymerase chain reaction

Total RNA was isolated from human NP cells using the TRIzol reagent (Invitrogen, USA) and was used to generate cDNA template for real-time PCR as described previously [19]. The expression of genes was determined by real-time PCR using ABI Prism 7500 (ABI, USA) and SYBR® Green Real-Time PCR Master Mix (TOYOBO, QPK-201). The primer sequences for RT–PCR are shown as follows: 5′-GAAATGAT-GGCTTATTACAGTGGC-3′ and 5′-GCCACTGTAATAAGCCATCATTTC-3′ for IL-1β; 5′-TCATCTACCCCAGGTCCTCTTCA-3′ and 5′-TGAAGAGGACCTGGGA-GTAGATGA-3′ for TNF-α; 5′-CTACCAGTGGATCGGCCTGAA-3′ and 5′-CGTGC-CAGATCATCACCACA-3′ for Aggrecan; 5′-CAGGTGAACCTGGACGAGAG-3′ and 5′-CCCACAGCACCAGTCTCAC-3′ for Collagen II; 5′-ATTCCATGGAGCCA-GGCTTTC-3′ and 5′-CATTTGGGTCAAACTCCAACTGTG-3′ for MMP-3; 5′-GCACCGTCAAGGCTGAGAAC-3′ and 5′-TGGTGA AGACGCCAGTGGA-3′ for GAPDH. All the primers were synthesized by TaKaRa (TaKaRa, China). The experiment was performed three times independently to obtain the mean value.

### ELISA

After each group of cells was treated, the supernatant of human NP cells was collected. The levels of TNF-α and IL-1β in cultured supernatant of NP cells were analyzed according to the instructions of the TNF-α and IL-1β ELISA kits. The experiment was performed three times independently to obtain the mean value.

### Fluorescence immunocytochemistry

Human NP Cells l were seeded in 2 × 10^5^ number on a 24‐well plate. NP cells in each group were treated and fixed with 4% paraformaldehyde for 10 min. The NP cells were sealed with normal goat serum for 1 h before being incubated with P65 antibody at 4 °C overnight. Finally, nuclear counterstaining was incubated with 4′6-diamidino-2-phenylindole and the cells were observed through a fluorescence microscope.

### Western blotting

Human NP cell protein was extracted with RIPA lysis buffer containing protease inhibitors. Proteins were resolved by 12% SDS/PAGE electrophoresis and transferred to a PVDF membrane (Millipore, Billerica, MA, USA). The membrane was blocked with 5% nonfat milk for 2 h and then probed with primary antibodies specific for P65, p-P65, aggrecan, collagen II, MMP-3 and MMP-9 (Cell Signaling Technology, USA) at 4 °C overnight. The experiment was performed three times independently to obtain the mean value.

### Statistical analysis

All data are presented as the means ± standard deviations (SD). The statistical analysis was assessed by the one‐way analysis of variance (ANOVA) followed by Student’s *t*-test. *P* values < 0.05 were considered statistically significant.

## Results

### Choosing an appropriate LIPUS intensity to inhibit of TNF-α and IL‐1β in LPS‐induced human NP cells

**Fig. 1:**
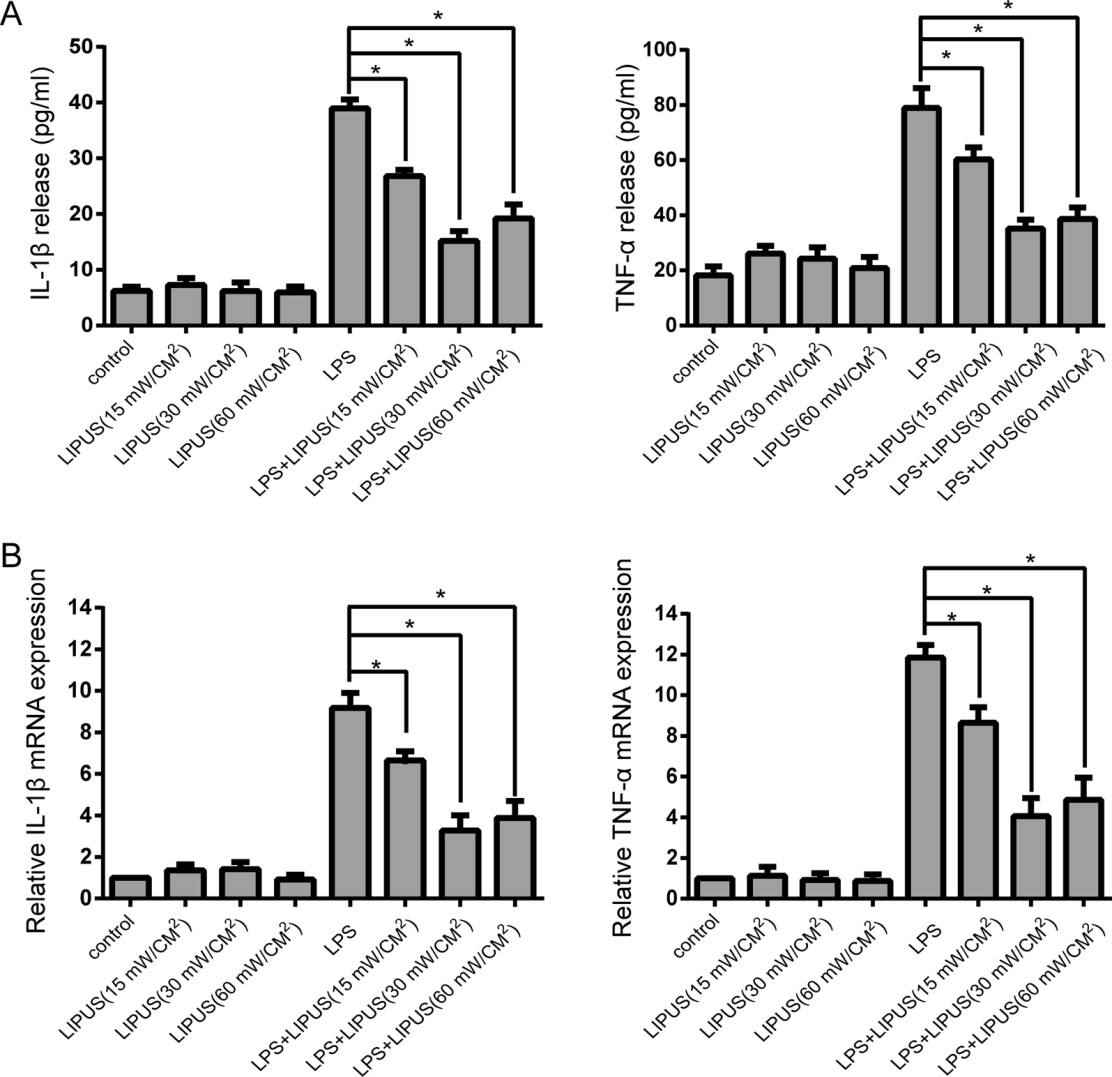
LIPUS inhibits the production and gene expression of the inflammatory factors TNF-α and IL‐1β in LPS‐induced human NP cells. **a** The protein expression of TNF-α and IL‐1β was measured by ELISA. **b** The mRNA expression of TNF-α and IL‐1β was measured by RT-PCR. The results are presented the means ± SD. *P < 0.05

To determine the effect of LIPUS on inflammatory factors in NP cells, ELISA (Fig. 1a) and RT-PCR (Fig. 1b) were used to measure the gene and protein expression of TNF-α and IL‐1β. In the LPS + LIPUS group, LIPUS intensities of 15 mW/cm^2^, 30 mW/ cm^2^ and 60 mW/cm^2^ significantly decreased the protein expression of TNF-α and IL‐1β compared with that in the LPS group (*P* < 0.05), and 30 mW/cm^2^ LIPUS showed the strongest inhibitory effect. In addition, 30 mW/cm^2^ LIPUS resulted in lower TNF-α and IL‐1β expression than 15 mW/cm^2^ or 60 mW/cm^2^ LIPUS (*P* < 0.05). Hence, we chose 30 mW/cm^2^ LIPUS as the appropriate LIPUS intensity to inhibit inflammatory factors for further experiments.

### LIPUS inhibits LPS-mediated catabolic effects in human NP cells

**Fig. 2:**
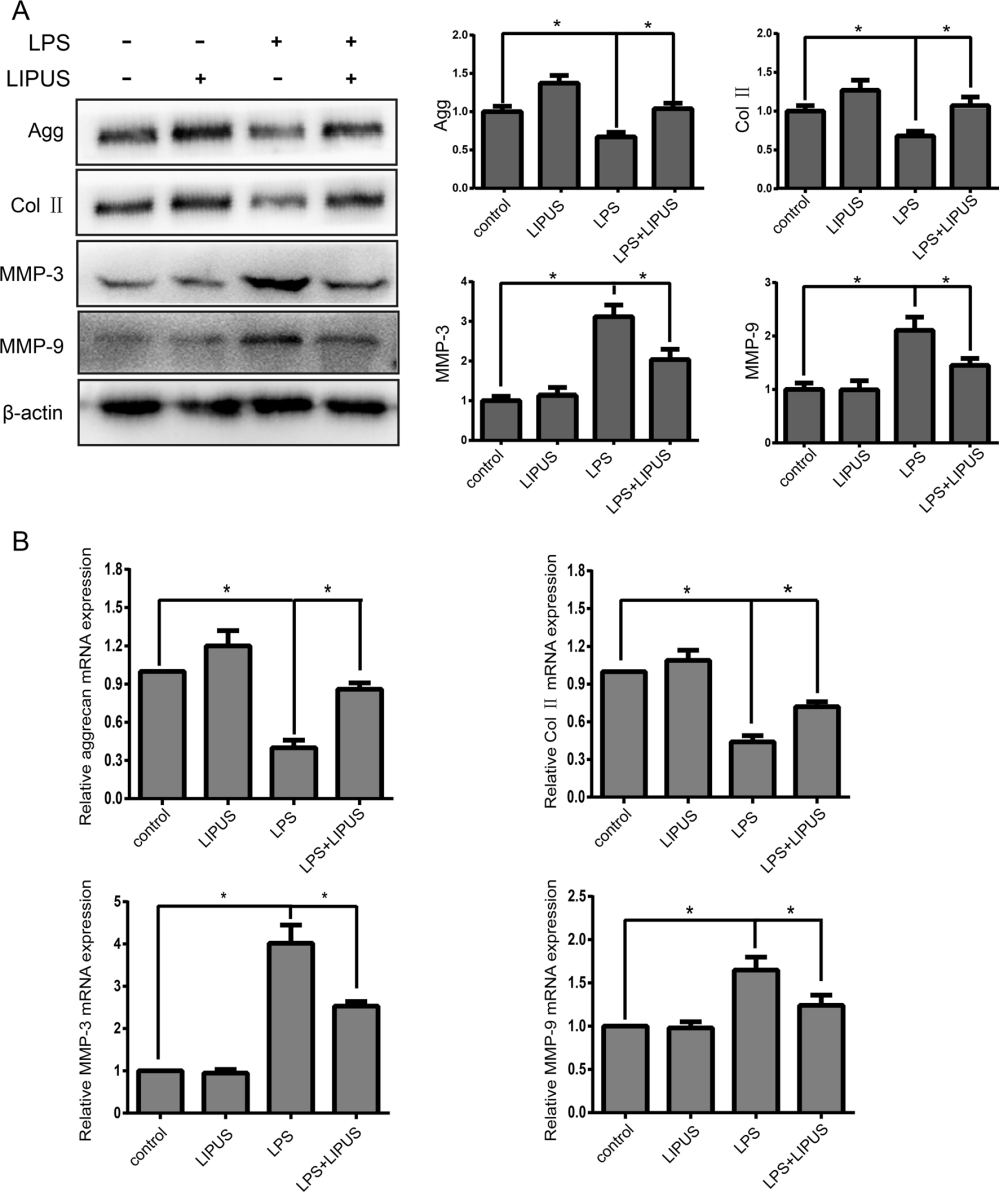
LIPUS inhibits LPS-mediated catabolic effect in human NP cells. **a** The protein expression of aggrecan, collagen II, MMP-3 and MMP-9 was measured by Western blot. **b** The mRNA expression of aggrecan, collagen II, MMP-3 and MMP-9 was measured by RT-PCR. The results are presented the means ± SD. *P < 0.05

To further clarify the effect of LIPUS on ECM metabolism, western blotting (Fig. 2a) and RT-PCR (Fig. 2b) were used to measure the gene and protein expression of aggrecan, collagen II, MMP-3 and MMP-9. LPS treatment significantly decreased the gene and protein expression of aggrecan and collagen II and increased the gene and protein expression of MMP-3 and MMP-9. However, LIPUS pretreatment significantly upregulated the gene and protein expression of aggrecan and collagen II and downregulated the gene and protein expression of MMP-3 and MMP-9. These results indicated that LIPUS exerted anti-inflammatory effects against ECM degradation by decreasing MMP expression.

### LIPUS inhibits the activation of NF‐κB in LPS‐induced human NP cells

**Fig. 3:**
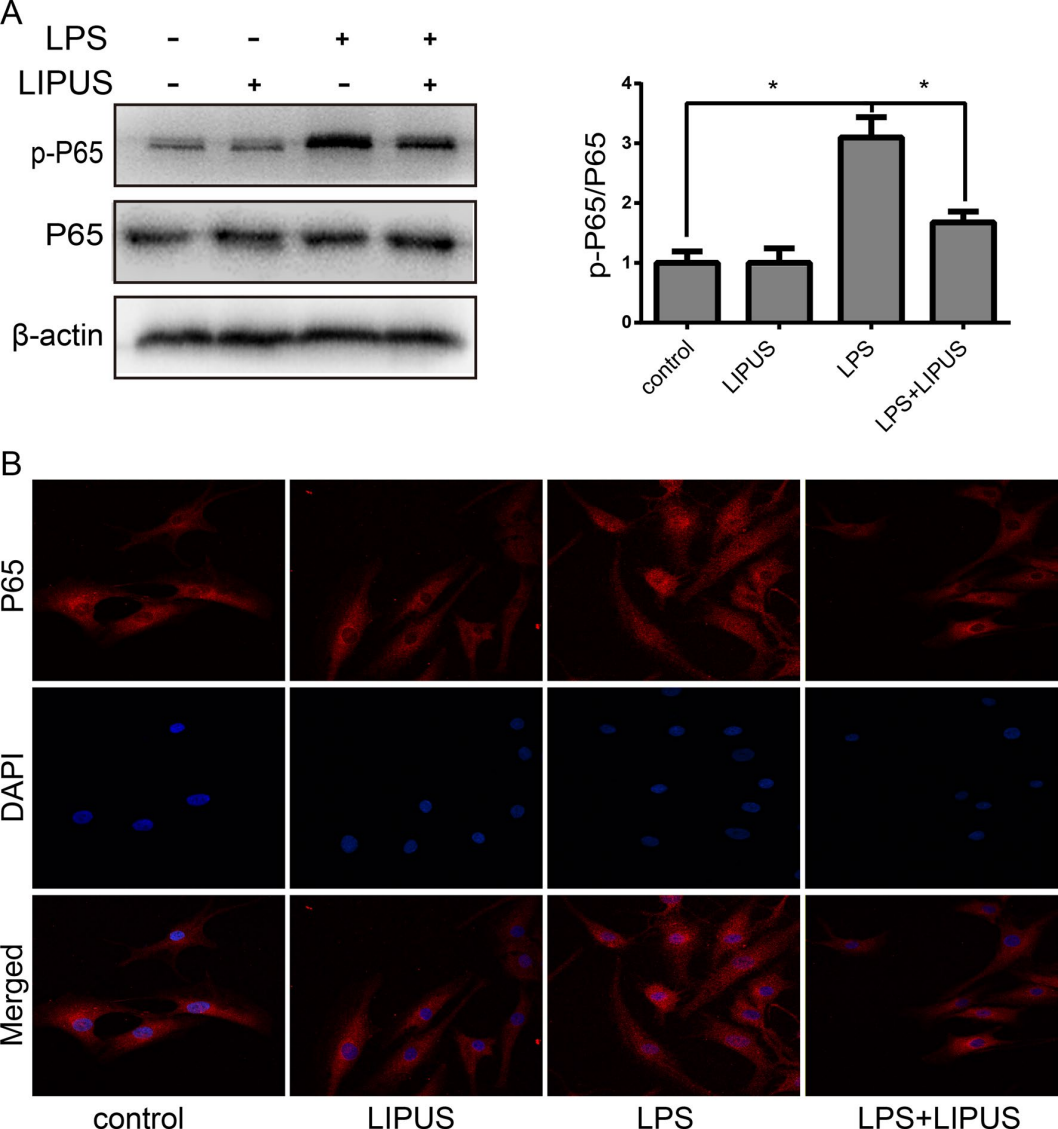
LIPUS inhibits the activation of NF‐κB in LPS‐induced human NP cells. **a** The protein expression of p-P65 and P65 was measured by Western blot. **b** The P65 nuclear translocation was detected by immunofluorescence. Magnification × 200. The results are presented the means ± SD. *P < 0.05

As the NF-κB signaling pathway has long been considered to be related to the regulation of inflammation, we investigated whether LIPUS could inhibit the secretion of inflammatory cytokines through this signaling pathway. LPS treatment significantly increased p-P65 protein levels, whereas LIPUS decreased p-P65 protein levels (Fig. 3a). Furthermore, immunofluorescence analysis was used to examine P65 nuclear translocation in NP cells. As shown in Fig. 3b, LIPUS inhibited the nuclear translocation of P65 in LPS-induced NP cells.

### LIPUS inhibits inflammation and catabolism through the NF‐κB pathway in LPS‐induced human NP cells

**Fig. 4:**
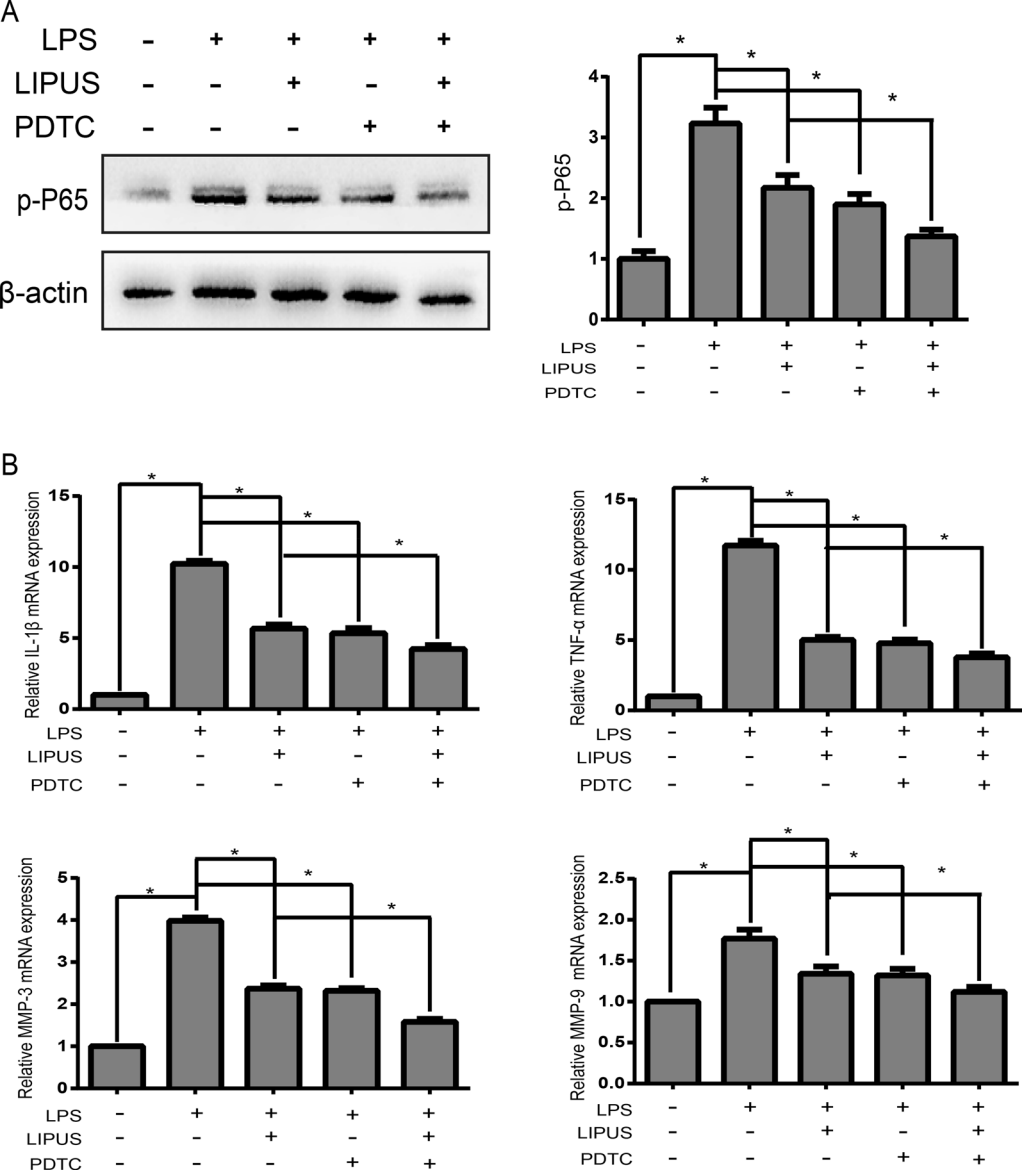
LIPUS inhibits inflammation and catabolism through the NF‐κB pathway in LPS‐induced human NP cells. **a** The mRNA expressions of TNF-α and IL-1β were measured by RT-PCR after PDTC pretreatment. **b** The mRNA expression of MMP-3 and MMP-9 was measured by RT-PCR after PDTC pretreatment. The results are presented the means ± SD. *P < 0.05

We further explored whether LIPUS played an anti-inflammatory and anti-catabolic role in LPS-induced NP cells through the NF-κB pathway. The NF‐κB pathway inhibitor, PDTC, was used to investigate the role of LIPUS. The western blot results showed that pretreatment with PDTC markedly decreased p-P65 protein levels (Fig. 4a). The RT-PCR results suggested that compared with that in the LPS group, the mRNA expression of the TNF-α, IL‐1β, MMP-3 and MMP-9 was decreased in the LPS + LIPUS and LPS + PDTC groups (Fig. 4b). In addition, the LPS + LIPUS + PDTC group exhibited a stronger synergistic effect in the reduction in gene expression than the LPS + LIPUS group. There was no significant difference between the LPS + LIPUS group and the LPS + PDTC group. Therefore, these results showed that LIPUS exerted anti-inflammatory and anti-catabolic effects on LPS-induced NP cells through the NF-κB pathway.

## Discussion

In the present study, we explored the effect of LIPUS on the inhibition of inflammation and catabolism in human degenerative nucleus pulposus cells. We observed that LIPUS could significantly inhibit the expression of the inflammatory factors TNF-α and IL-1β and the production of the matrix metalloproteinases MMP-3 and MMP-9 and promote the synthesis of the extracellular matrix aggrecan and collagen II in LPS-induced NP cells.

LIPUS is a noninvasive mechanical treatment option with potential regenerative effects and is widely used in clinical practice to relieve soft tissue injuries and accelerate bone fracture healing [19, 20]. In our previous study, LIPUS protected against cartilage degeneration in experimental osteoarthritis [21]. However, studies on the relationship between LIPUS and intervertebral disc degeneration only show that LIPUS can promote the synthesis of NP cell extracellular matrix, but the role of LIPUS on inflammation in intervertebral disc degeneration and its molecular mechanism has not yet been clarified. Zheng et al. reported that LIPUS inhibited the LPS-induced inflammatory response in RAW264.7 macrophages [22]. Zhang et al. also reported that LIPUS alleviated the expression of the inflammatory factors TNF-α, IL-1β, IL-6 and IL-8 induced by LPS [23]. In our study, we also observed a similar result in human NP cells: LIPUS inhibited the expression of the inflammatory factors TNF-α and IL-1β in LPS-induced human NP cells. In addition, we also found that LIPUS intensities of 15 mW/cm^2^, 30 mW/ cm^2^ and 60 mW/cm^2^ significantly decreased the gene and protein expression of TNF-α and IL‐1β. Moreover, the 30 mW/cm^2^ LIPUS was the most effective intensity for decreasing TNF-α and IL‐1β expression.

Several investigations have shown that the inflammatory factors IL-1β and TNF-α contribute to IVDD by inhibiting ECM production and upregulating catabolic enzymes [24, 25]. However, whether LIPUS could reverse the extracellular matrix metabolism of LPS-induced NP cells was unclear. Mengtong Guan et al. found that LIPUS reduced the expression of catabolic genes in IL-1β-treated primary murine chondrocytes. Similarly, the same results were observed in our study: LIPUS significantly increased the production of ECM (aggrecan and collagen II) and inhibited the production of matrix metalloproteinases (MMP-3 and MMP-9) in LPS-induced human nucleus pulposus cells.

To further explore the possible mechanism of LIPUS involved in LPS-induced NP cells, we also examined the NF-κB signaling pathway in this study. NF-κB is a nuclear transcription factor that plays an important role in regulating cell proliferation, inflammatory response, apoptosis and matrix production. Under normal conditions, NF-κB mainly exists in the cytoplasm as a trimer with the inhibitors IκB and P65/P50. When activated, IκB is phosphorylated and degraded, and P65 enters the nucleus from the cytoplasm. It has been reported that LIPUS suppresses LPS-induced IL-1α by inhibiting NF-κB nuclear translocation in MC3T3-E1 cells [26]. Chen et al. also reported that LIPUS attenuated LPS-induced neuroinflammation by modulating TLR4/NF-κB signaling way [27]. In addition, a previous study reported that an NF-κB inhibitor attenuated intervertebral disc degeneration [28]. The PPAR-γ agonist pioglitazone inhibits IL-17-induced intervertebral disc inflammation and degeneration through the NF-κB signaling pathway [29]. Our findings also showed that LIPUS inhibited p-P65 protein expression and NF-κB nuclear translocation in LPS-induced NP cells. Therefore, the anti-inflammatory and anti-catabolic effects of LIPUS on LPS-induced NP cells likely occur through the NF-κB pathway.

**Fig. 5:**
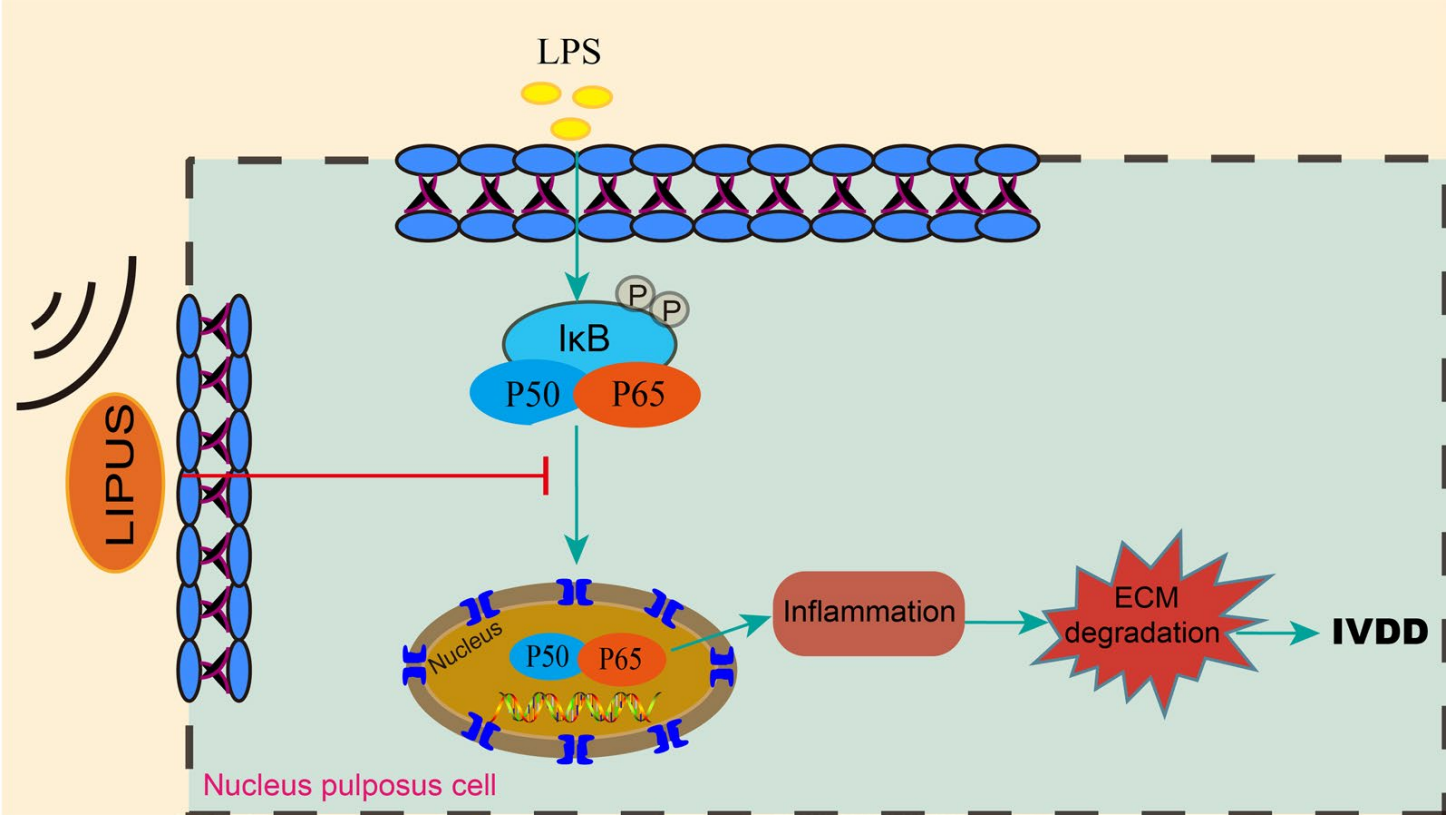
Schematic diagram illustrating the mechanism of LIPUS on inflammation and catabolic effect via the NF-κB pathway in LPS-induced human nucleus pulposus cells

In summary, our findings revealed that LIPUS inhibited the expression of the inflammatory cytokines IL-1β and TNF-α and increased the production of ECM (aggrecan and collagen II) and inhibited the production of matrix metalloproteinases (MMP-3 and MMP-9) in LPS-induced human NP cells, which may be through the NF-κB pathway (Fig. 5). These results reveal the therapeutic effect of LIPUS on intervertebral disc degeneration, which will provide theoretical support for the application of LIPUS in the clinical treatment of intervertebral discs. It is necessary to conduct further research to clarify the mechanism of LIPUS inhibiting inflammation and catabolism through the NF-κB signaling pathway.

## Data Availability

The datasets used and/or analyzed during the current study are available from the corresponding author on reasonable request.

## Abbreviations

TNF-α: Tumor necrosis factor-α
IL-1β: Interleukin-1β
CCK-8: Cell count kit-8
NP: Nucleus pulposus
LIPUS: Low-intensity pulsed ultrasound
MMP: Matrix metalloproteinase
IVDD: Intervertebral disc degeneration
PVDF: Polyvinylidene difluoride
ANOVA: One-way analysis of variance
